# Omalizumab: Practical considerations regarding the risk of anaphylaxis

**DOI:** 10.1186/1710-1492-6-32

**Published:** 2010-12-03

**Authors:** Harold L Kim, Richard Leigh, Allan Becker

**Affiliations:** 1 University of Western Ontario, London, Ontario, Canada and McMaster University, Hamilton, Ontario, Canada; 2 Departments of Medicine and Physiology & Pharmacology, Snyder Institute of Infection, Immunity and Inflammation, University of Calgary, Calgary, Alberta, Canada; 3 Section of Allergy and Clinical Immunology, Department of Pediatrics and Child Health, University of Manitoba, Winnipeg, Manitoba, Canada

## Abstract

Omalizumab has demonstrated efficacy among patients with moderate to severe persistent allergic asthma, whose symptoms are inadequately controlled with other controller agents. This therapy is generally well tolerated, but there are some safety considerations, the most important of which is the rare, but potentially life-threatening, occurrence of omalizumab-associated anaphylaxis.

In Canada, data from the manufacturer of omalizumab indicate that the frequency of anaphylaxis attributed to Xolair in post-marketing use is approximately 0.2%. Other researchers, including the American Omalizumab Joint Task Force (OJTF), have suggested a lower overall frequency of 0.09%.

This paper provides a summary of the epidemiologic research carried out to date and presents a concise, practical set of recommendations for the prevention, monitoring and management of omalizumab-associated anaphylaxis. Prevention tips include advice on patient education measures, concomitant medications and optimal administration. For the first three injections, the recommendation is to monitor in clinic for two hours after the omalizumab injection; for subsequent injections, the monitoring period should be 30 minutes or an appropriate time agreed upon by the individual patient and healthcare professional.

In the event that a patient does experience omalizumab-associated anaphylaxis, the paper provides recommendations for handling the situation in-clinic and recommendations on how to counsel patients to recognize the potential signs and symptoms in the community and react appropriately.

## Introduction

Omalizumab, a recombinant humanized monoclonal anti-IgE antibody, is indicated for patients with moderate to severe persistent allergic asthma, whose symptoms are inadequately controlled with high-dose inhaled corticosteroids either alone or in combination with a long-acting β_2_-agonist [1-3]. This compound has demonstrated efficacy in this patient population in a number of clinical studies [4-14], and its use for severe allergic asthma has been endorsed by several Canadian and International consensus bodies [2,3,15-18]. According to the 2010 Canadian Thoracic Society's Asthma Management Continuum, omalizumab can be used for "patients with difficult-to-control asthma confirmed with objective measures, who have documented allergies to a perennial aeroallergen, a serum IgE level of 30 IU/mL to 700 IU/mL and whose asthma symptoms remain uncontrolled despite adherence to high-dose inhaled corticosteroids plus at least one additional controller therapy [16]."

**Figure 1:**
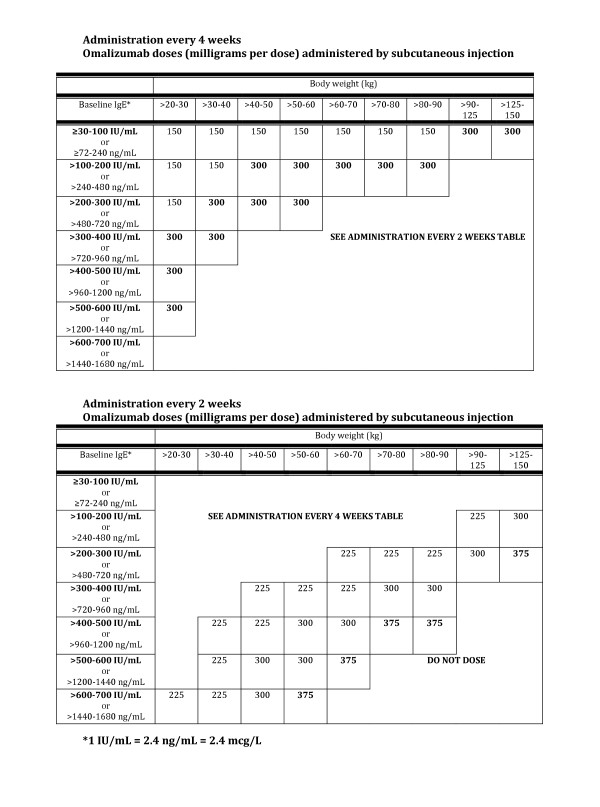
**Dosing of Omalizumab by Body Weight and Baseline IgE**.

Omalizumab is administered as a subcutaneous injection, once every two or four weeks. The dosage is dependent on body weight and the serum IgE level (Figure 1) [1]. While this therapy is generally well tolerated, there are some safety considerations. The most important of these is the rare, but potentially life-threatening, occurrence of anaphylaxis, which has been shown to occur in < 0.1% of patients treated with omalizumab.

This review will discuss the variable presentation of anaphylaxis associated with omalizumab, consider the mechanisms involved in omalizumab-associated anaphylaxis, present the most recent incidence data and provide practical recommendations regarding patient education, monitoring and treatment.

## Definition and presentation of omalizumab-associated anaphylaxis

Perhaps the best definition of anaphylaxis is that proposed by a joint venture of the American National Institute of Allergy and Infectious Disease (NIAID) and the Food Allergy and Anaphylaxis Network in 2006. They defined anaphylaxis as a reaction "with skin or mucosal involvement, airway compromise and/or reduced blood pressure with or without associated symptoms, and a temporal relationship to allergen exposure [19]."

Anaphylaxis related to omalizumab has been described as a combination of any of the following: angioedema of the throat or tongue, bronchospasm, hypotension, syncope, and/or urticaria [1].

## Mechanism of anaphylaxis with omalizumab

At the present time, there is no consensus regarding the mechanism(s) underlying omalizumab-associated anaphylaxis. There have, however, been several hypotheses proposed. These include a potential pre-existing anti-allotypic or anti-idiotypic antibody (IgE or IgG) against omalizumab. Alternatively, such an antibody may possibly develop after initial exposure or as a response to cumulative exposure to the drug [20].

There is also the possibility that polysorbate, one of the formulation's excipients, is responsible for anaphylactic reactions [21,22]. This additive is used to enhance the solubility of the drug in the aqueous solution. Previous research has shown that it may be associated with hypersensitivity reactions when used in formulations of erythropoietin or darbopoietin [21]. Investigation into two anaphylactic reactions to omalizumab also concluded that it was the polysorbate component of the formulation that was responsible for these particular reactions [22].

Another hypothesis is that these events may, in some patients, be unrelated to the drug itself. Many patients who receive omalizumab will also be receiving concomitant immunotherapy. Indeed, there is evidence that adding omalizumab to immunotherapy is more effective than immunotherapy alone among children with seasonal allergic rhinoconjunctivitis and co-morbid seasonal allergic asthma [23]. Should anaphylaxis occur in these patients, as has been reported in the literature [24], it is more likely that the reaction is due to the immunotherapy rather than the omalizumab.

Finally, there is also the possibility that an anaphylactic reaction may be attributable to exposure to another allergen (*e.g*., ingested food) around the time of the omalizumab administration [20].

## Incidence of anaphylaxis with omalizumab

In Canada, the manufacturers of omalizumab (Novartis Pharmaceuticals Canada, Inc.) administer a physician and patient support program, known as the Xolair Healthcare Assistance and Link to Education (XHALE), which assists with administration of the compound at local specialized clinics, patient education, dispensing and reimbursement. This program also compiles data on compliance.

The most recent data available from XHALE (June, 2010) show that the incidence of omalizumab-associated anaphylaxis in Canada is similar to the rate of anaphylaxis that is reported in the product monograph [21].

There are a number of other sources that have quantified the incidence of omalizumab-associated anaphylaxis. In the pre-marketing, clinical-trial period, the incidence was found to be approximately 0.08% (3 of 3854 patients) [1]. Subsequent to the agent's availability for clinical use, the drug's manufacturer reported the incidence of anaphylaxis to be "at least 0.2%", based on an approximate sample size of 57,300 patients who had taken the drug between June 2003 and December 2006. This estimated incidence was based on spontaneous reports.

In 2006, an independent body endorsed by the American Academy of Allergy, Asthma & Immunology and the American College of Allergy, Asthma and Immunology, known as the Omalizumab Joint Task Force (OJTF), convened to examine omalizumab-associated anaphylaxis [20]. This body published a review, including a set of recommendations, in December, 2007.

The OJTF examined post-marketing reports compiled by the agent's manufacturers and, using the above definition of anaphylaxis [19], concluded that there were 41 episodes among 35 patients that could reasonably be defined as anaphylaxis. During the period of review, there were 39,510 patients being treated with omalizumab. This corresponds to an overall incidence of approximately 0.09% [20]. There were no fatalities associated with these episodes and none of the patients required intubation.

Omalizumab-associated anaphylaxis typically occurs within the first two hours after injection. The OJTF report observed that 16 of the 41 identified episodes of anaphylaxis (39%) occurred within 30 minutes, with a further 12 episodes occurring between 30 minutes and two hours post-dose. Combined, 28 of the 41 episodes (68%) occurred within the first two hours. Five episodes occurred between two and 12 hours post-dose and three episodes occurred more than 12 hours after dosing. There were also five episodes with unknown timing (with respect to time elapsed). Removing these five episodes from the analysis, 78% of the remaining episodes occurred within the first two hours after injection (28/36 episodes) (Table 1).

**Table 1:** Timing of omalizumab-associated anaphylaxis*

Timing of the reaction	Number of Episodes		
	
	1^st^, 2^nd ^or 3^rd ^Omalizumab dose, n	4^th ^Omalizumab dose or later, n (%)	Total
< 30 minutes	11	5	16
30 - 60 minutes	6	1	7
1 - 2 hours	5	0	5
**Total 0 - 2 hours**	**22**	**6**	**28**
2 - 12 hours	4	1	5
> 12 hours	3	0	3
**Overall Total**	**29**	**7**	**36**

*Data represent 36 of 41 identified episodes of anaphylaxis; timing was not known for the remaining 5 episodes. The 41 episodes were among 35 patients. During the period of review, there were 39,510 patients being treated with omalizumab.

There have been several published case reports of patients whose anaphylactic reaction fell outside the two-hour post-dose window [20,25-27]. For example, one published account described a woman who experienced throat irritation, pruritus of her ears, and wheeze requiring use of inhaled salbutamol, two and a half hours following her first omalizumab injection [27].

Most reactions do occur within the first several injections. Of the 41 episodes identified by the OJTF, 32 (78%) occurred within the first three injections. There is, however, still a small risk of later-onset anaphylaxis. For example, a patient who had been receiving omalizumab for 14 months experienced an anaphylactic reaction on her 27^th ^injection, which resolved with treatment in the office [28]. Other cases occurring after more than a year of successful omalizumab treatment have also been reported [23].

## Recommendations

### Patient education

All patients who are candidates for omalizumab therapy should be informed of both the potential benefits of the medication and also of the possibility of rare adverse events. In particular, patients should receive counseling about the potential symptoms and signs of anaphylaxis and an explanation that the potential for such events is the motivation behind post-dose monitoring. See Appendix 1 for a sample patient letter regarding the benefits and risks of omalizumab.

### Informed consent

Subsequent to the provision of education regarding the benefits and potential risks of omalizumab therapy, patients should be asked to provide signed, informed consent prior to receiving omalizumab treatment injections, which should then be entered into his or her medical record.

### Review of medications

Before initiating omalizumab therapy, one should review the patient's medications to ensure he or she is not taking any medication that could interfere with rescue epinephrine therapy. The concomitant use of beta-blockers should be discouraged during omalizumab therapy for this reason. Patients should continue to take other asthma medications unless the regimen is changed by the managing physician.

### Administration

The recommended steps for proper reconstitution and administration of omalizumab (as well as the materials required) are shown in Table 2. While not all practitioners are familiar with the process, the directions are straightforward and simple to learn. To determine the dose of omalizumab to administer, consult the easy-to-use dosing tables in the product's prescribing information (Figure 1).

**Table 2:** Reconstitution and administration of omalizumab.

**Step 1:**	Draw 1.4 mL of SWFI, USP into a 3-cc syringe equipped with a 2.5 cm, 18 gauge needle.
**Step 2:**	Place the vial upright on a flat surface and using standard aseptic technique, insert the needle and inject the SWFI, USP directly onto the product.
**Step 3:**	Keeping the vial upright, gently swirl the upright vial for approximately 1 minute to evenly wet the powder. Do not shake.
**Step 4:**	After completing Step 3, gently swirl the vial for 5-10 seconds approximately every 5 minutes in order to dissolve any remaining solids. There should be no visible gel like particles in the solution. Do not use if foreign particles are present.*
**Step 5:**	Invert the vial for 15 seconds in order to allow the solution to drain toward the stopper. Using a new 3-cc syringe equipped with a 2.5 cm, 18 gauge needle, insert the needle into the inverted vial. Position the needle tip at the very bottom of the solution in the vial stopper when drawing the solution into the syringe. Before removing the needle from the vial, pull the plunger all the way back to the end of the syringe barrel in order to remove all of the solution from the inverted vial.
**Step 6:**	Replace the 18 gauge needle with a 25 gauge needle for subcutaneous injection.
**Step 7:**	Expel air, large bubbles, and any excess solution in order to obtain the required 1.2 mL dose. A thin layer of small bubbles may remain at the top of the solution in the syringe. Because the solution is slightly viscous, the injection may take 5 to 10 seconds to administer.

* Some vials may take longer than 20 minutes to dissolve completely. If this is the case, repeat Step 4 until there are no visible gel like particles in the solution. It is acceptable to have small bubbles or foam around the edge of the vial. Do not use if the contents of the vial do not dissolve completely by 40 minutes.

With the risk of treatment associated anaphylaxis subsequent to omalizumab administration, the setting for administration is also important. This agent should only be administered by a physician or other licensed health care professional, who is trained in the recognition and treatment of anaphylaxis, and should only be administered in a setting where the appropriate medications and equipment are available to respond to an episode of anaphylaxis.

At the time of each administration, the healthcare professional should assess the patient's current health, vital signs and asthma control, to ensure that there have been no recent changes that might affect the decision of whether or not to administer omalizumab that day. A sample of a standardized assessment sheet can be found in Table 3. Spirometry is not indicated at every visit, but may be performed at regular intervals (e.g., every three months) or when clinically indicated.

**Table 3:** Sample Omalizumab Patient Assessment Sheet

Patient Information		
Patient name:	DOB:	

Date of visit:	Location of administration:	

Omalizumab dose:	Date of first omalizumab administration:	

# of prior omalizumab injections:	Last omalizumab administration date:	

**Pre-administration Evaluation**		

Blood pressure:	Respiratory rate:	

Pulse:	Temperature:	

**Asthma control questionnaire**		

How many times per week do you have asthma symptoms during the day?		

How many times per week do you have asthma symptoms at night?		

Has your asthma affected your ability to perform physical activities?		

How many asthma attacks have you had in the past week? Month?	per week: __________	per month: _________

Has your asthma caused you to miss any work/school?		

How many times per week do you have to use your rescue inhaler?		

**Spirometry results (if indicated)**		

FEV_1_	FVC:	

**Other results**		

		

**Post administration information**		

Duration of post-administration observation		

Note any adverse reactions here:		

### Monitoring

Because of the risk of anaphylaxis, patients should be closely observed for an appropriate period of time after omalizumab administration. The data detailed above show that if a patient does not experience anaphylaxis during the first two hours and first three injections, it is considerably less likely that he or she will ever experience anaphylaxis. However, cases have been described in the literature where anaphylactoid events have occurred several hours after injection. If one accepts the OJTF's estimate of an overall incidence of 0.09% (i.e., less than 1 case per 1,000 patients treated), and the OJTF's finding that 78% of episodes occur in the first two hours, the overall incidence of an anaphylactic reaction occurring later than that is approximately 1 in 4,000 to 5,000 patients. Given that the OJTF also reported that 32 of the 41 episodes (78%) occurred within the first three injections, the likelihood of anaphylaxis occurring in a fourth or subsequent injection is therefore of a similar magnitude (*i.e*., 0.023% incidence, or approximately 1 in 4,000 injections).

With these statistics in mind, the recommended period of monitoring should be two hours following the first three injections. For subsequent injections, when the risk of anaphylaxis is substantially lower, the observation period can be significantly reduced. The suggested period for monitoring is 30 minutes, but this may be adapted for individual patients following discussion between the patient and the healthcare professional of the continued risk of anaphylaxis. Should patients be unwilling to remain in clinic for the physician-recommended period of monitoring, they should be asked to sign a waiver indicating their preference and abrogating the physician and manufacturer from any responsibility relating to potential anaphylactic events during that time. Physicians need to consider whether they will agree to continue to provide care to such patients.

### Treatment in clinic

Registered nurses or physicians administering omalizumab should be prepared and trained to manage episodes of omalizumab-induced anaphylaxis. Clinics administering omalizumab should have resuscitation equipment available in the clinic; this equipment should be checked and updated on a regular basis. In situations where a registered nurse administers the injection, a physician experienced in the management of acute anaphylaxis should also be available in the immediate vicinity.

The steps to be taken in the event of an anaphylactic reaction in the clinic are discussed below in the summary of recommendations. The initial assessment should include airway, breathing and circulation. Epinephrine (0.3 mg intramuscularly) should be injected in the lateral thigh, and emergency medical services should be contacted. The epinephrine should be repeated if the symptoms are worsening or not improving over the next 5-10 minutes.

The patient should then be placed in a recumbent position, with the lower extremities elevated (if tolerated). Patency of the airway must be continuously monitored and maintained. If the symptoms are severe, the administration of oxygen is recommended, and venous access should be established, with the line kept open with normal saline.

With respect to rescue medication, one can consider the use of bronchodilators if necessary (*e.g*., salbutamol MDI or nebulized 2.5 - 5 mg in 3 mL saline). Additional medications (*e.g*., H_1 _antihistamines or systemic corticosteroid) may also be administered according to clinical judgment.

### Treatment in the community

At the time the decision is made to initiate omalizumab therapy, the patient should be given explicit instruction on what to do should he or she experience signs or symptoms of anaphylaxis subsequent to the in-clinic monitoring period. These verbal instructions should be accompanied by a clearly written patient information hand-out. Patients must have an epinephrine auto-injector and be instructed on its use. After treating with the epinephrine, patients should then proceed to the nearest emergency room.

Omalizumab should be administered in the clinical setting; to date, there is no precedent for widespread home administration. There is, however, a small observational study (n = 25) that suggests omalizumab may be effectively and safely self-administered in the home [29]. Further research is required before this can be considered a viable option.

Importantly, regardless of where anaphylaxis occurs, omalizumab's product monograph indicates that the agent should be permanently discontinued in any patients who experience a severe hypersensitivity reaction.

### Summary of recommendations

Patient education:

1) Provide counseling about the benefits of the medication and the possibility of rare adverse events;

2) Discuss the potential signs and symptoms of anaphylaxis, reinforce with take-home handout;

3) Explain how the risk is reduced as time elapses post-dose and as the number of doses increases;

4) Link relative incidence of anaphylaxis to the duration of post-dose monitoring; and

5) Obtain written, informed consent and include in the medical record

6) Give explicit instruction on what to do when signs or symptoms of anaphylaxis are experienced in the community; and

7) Ensure the patient receives an epinephrine auto-injector and is instructed on its use

Medications:

1) Where feasible, discontinue beta-blocker therapy before initiating omalizumab; and

2) Patients should continue to take other asthma medications unless the regimen is changed by the managing physician.

Administration:

1) Administer in a setting where the appropriate medications and equipment are available to respond to an episode of anaphylaxis;

2) Omalizumab should only be administered by a physician or registered nurse who is trained in the recognition and treatment of anaphylaxis; and

3) At each administration, health, vitals and asthma symptoms should be assessed.

### Monitoring

1) For the first three injections: Monitor in clinic for two hours after the omalizumab injection. Reinforce patient education regarding signs, symptoms and how to treat in the community;

2) For the fourth and subsequent injections, monitor of 30 minutes, or for an appropriate time agreed upon by the individual patient and healthcare professional; and

3) If the patient refuses to wait for the recommended period of time, he or she must sign a waiver.

Treatment in clinic:

1) Assess airway breathing and circulation;

2) Inject epinephrine, 0.3 mg i.m., in the lateral thigh;

3) Epinephrine dosing should be repeated if necessary;

4) Contact emergency services;

5) Establish, monitor and maintain patency of the airway;

6) Place patient in recumbent position, with elevated lower extremities, if tolerated;

7) Administer oxygen;

8) Establish venous access with an i.v. line; keep open with normal saline;

9) Consider use of short-acting bronchodilator (*e.g*., salbutamol); and

10) Consider additional medications (e.g., H1 antihistamine, systemic corticosteroid).

Treatment in

1) Treat with autoinjection of epinephrine; and

the community

2) Proceed to the nearest emergency room/contact emergency services.

Post-reaction:

3) Permanently discontinue omalizumab in any patient who experiences a severe hypersensitivity reaction.

## Discussion

Healthcare professionals should keep the risk for omalizumab-associated anaphylaxis in perspective; rare, serious adverse events are an unfortunate risk of many effective medications across all medical specialties. In the risk: benefit analysis, the very small risk of experiencing a serious adverse event needs to be weighed against the potential significant benefits of employing that therapy.

Omalizumab is not the only agent that has been associated with a risk of anaphylaxis. Indeed, anecdotally, the risk of anaphylaxis associated with omalizumab appears to be lower than the risk associated with immunotherapy for allergy [30]. There are a number of common therapies that are routinely administered to outpatients that have also been associated with such a risk. These include antibiotics (particularly penicillin), aspirin, non-steroidal anti-inflammatory drugs (NSAIDS; e.g., diclofenac) and opioid analgesics [31]. The reported incidence of penicillin hypersensitivity is between 1% and 10% [32]. Aspirin hypersensitivity is extremely common among patients with asthma, affecting as many as 15% of these patients [33].

Another example of a well-known rare and serious adverse event associated with an effective medication is ACE-inhibitor-associated angioedema. The overall incidence of angioedema associated with this class of antihypertensive medications (*e.g*., ramipril, enalapril) is comparable to that of omalizumab-associated anaphylaxis: approximately 0.1% [34]. Despite this well known risk, however, ACE inhibitors are among the most widely prescribed medications in Canada and around the world.

Omalizumab is a valuable therapy that can provide control of asthma symptoms to patients with allergic-mediated asthma who have been unable to achieve control on traditional inhaled and/or oral controller medications. The risk-benefit equation for omalizumab comes out strongly in favor of the benefit for the majority of appropriately selected patients who receive it. It is also worth noting that the alternative to omalizumab in these difficult-to-treat patients is usually oral corticosteroids, which are associated with major, serious and well known adverse events (*e.g*., increased risk of osteoporosis, cardiovascular disease, hyperglycemia, cataracts and glaucoma) [35]. Even among those patients who do experience a hypersensitivity reaction, one should consider the possibility of re-instating omalizumab therapy on a case-by-case basis. Some patients may be able to resume omalizumab therapy at a lower dose or through the use of a desensitization procedure.

Persistent, severe asthma can have a devastating impact on a patient's quality of life and the treatment of these patients represents a significant share of asthma-related health-care expenditures. For patients who are candidates for omalizumab therapy, clinicians should be comfortable prescribing this medication. Adequately informed and educated patients who choose to accept this therapy and are monitored appropriately and treated according to the recommendations contained in this document are at negligible risk from omalizumab-associated anaphylaxis.

## Competing interests

Dr. Harold Kim is the president of the Canadian Network for Respiratory Care and co-chief editor of Allergy, Asthma and Clinical Immunology. He has received consulting fees and honoraria for continuing education from AstraZeneca, GlaxoSmithKline, MerckFrosst, Novartis, and Nycomed.

Dr. Richard Leigh is a CIHR Clinician-Scientist (Phase 2), an AHFMR Clinician Investigator, and holds the GlaxoSmithKline-CIHR Professorship in Inflammatory Lung Disease. He has received consulting fees from AstraZeneca Canada, GlaxoSmithKline, Novartis Pharmaceuticals Canada, and Boehringer-Ingelheim Canada, and is on the speakers' bureau for AstraZeneca Canada, GlaxoSmithKline, and Novartis Pharmaceuticals Canada. In addition he has received research support from AstraZeneca, Ception, Genentech, GlaxoSmithKline, Novartis, MedImmune and MerckFrosst.

Dr. Allan Becker is a member of advisory boards for AstraZeneca, MerckFrosst and Novartis. He has received honoraria for continuing education from AstraZeneca, Graceway, MerckFrosst and Nycomed. He has received research support from AstraZeneca, GlaxoSmithKline, MerckFrosst, Novartis and Nycomed. His primary research support is from CIHR, the AllerGen NCE and NSERC.

## Authors' contributions

HK contributed to the drafting and writing of the manuscript and to revising it for important intellectual content; as such, he has given final approval of the version to be published.

RL contributed to the drafting the manuscript and to revising it critically for important intellectual content; as such, he has given final approval of the version to be published.

AB contributed to revising the manuscript critically for important intellectual content; as such, he has given final approval of the version to be published.

## Appendix 1. Sample Patient Letter

### Patient information letter: Xolair (omalizumab)

#### What is Xolair (omalizumab), and why is it being prescribed?

The allergic person makes too much of a certain protein in the body, called IgE antibody. The overproduction of this protein may result in the development of various allergic conditions such as allergic rhinitis (hay fever), allergic asthma, venom sensitivity, food or drug allergy.

Xolair is a drug that acts by binding to the IgE allergic antibody in the blood stream and blocking its actions. Health Canada has approved Xolair for the treatment of patients with moderate to severe persistent asthma. Xolair is used for adults and adolescents (12 years of age and above) who have a positive skin or laboratory test confirming allergy and whose symptoms are inadequately controlled with inhaled corticosteroids.

#### Benefits of Xolair

Xolair has been shown to decrease the number of asthma attacks in patients with moderate to severe asthma, and in some patients it allows a reduction in other asthma medications.

#### How is it given, how often is it given, and for how long?

Your Xolair dose will be chosen based on your body weight and the results of a blood test that measures your level of IgE. You will receive 1-2 injections of Xolair in your upper arm every 2 to 4 weeks depending on these factors. Unless your weight changes significantly, the dose and injection schedule should not change once your treatment has started.

It may take several months before you begin to notice benefits from Xolair. However, once benefits are observed, they should last for as long as you continue to receive your regular injections. If for some reason your injections are stopped, we would expect the effects to wear off within 6 months to a year.

#### What are the risks associated with its use?

The clinical studies performed for the approval of this medication suggest that Xolair is very safe. The overall number of adverse reactions has been similar among those patients taking Xolair or placebo (an inactive ingredient). These adverse reactions have included injection site reactions (45%), colds (23%), sinus infections (16%), headache (15%), and sore throat (11%)

Serious adverse reactions occurred in less than 1% of patients. The most serious reactions occurring in studies with Xolair were generalized allergic reactions (anaphylaxis) from receiving the drug.

#### Generalized allergic reactions (anaphylaxis) and their treatment

Anaphylaxis has been noted to occur within 2 hours of the first or subsequent dose of Xolair in a small minority (< 0.1%) of study volunteers without other identifiable allergic triggers. The reactions included hives and throat and/or tongue swelling. At the first sign of a generalized allergic reaction, adrenaline (epinephrine) is usually given to counteract the reaction.

#### Local reactions and their treatment

Local reactions that consist of swelling of the arm, redness or tenderness at the site of injection are usually handled with simple measures such as local cold compresses or the use of medications such as antihistamines or aspirin.

#### Where will the injections be administered?

Since the possibility exists that a Xolair injection may cause a generalized allergic reaction, we require that Xolair be administered at a facility equipped to treat you if you experience such a reaction. You will be observed up to two hours after each injection for the first three injections. The period of observation will be determined based on your physician's instructions. A doctor who can treat severe reactions to the drug will be available in the clinic during the time that you are present.

If you develop a delayed reaction to your Xolair injection (after you leave our facility) please either return to our Center or proceed to the nearest emergency room and then contact us as soon as possible.
